# Adaptive trust calibration for human-AI collaboration

**DOI:** 10.1371/journal.pone.0229132

**Published:** 2020-02-21

**Authors:** Kazuo Okamura, Seiji Yamada

**Affiliations:** 1 Department of Informatics, School of Multidisciplinary Sciences, The Graduate University for Advanced Studies (SOKENDAI), Tokyo, Japan; 2 Digital Content and Media Sciences Research Division, National Institute of Informatics, Tokyo, Japan; Nanyang Technological University, SINGAPORE

## Abstract

Safety and efficiency of human-AI collaboration often depend on how humans could appropriately calibrate their trust towards the AI agents. Over-trusting the autonomous system sometimes causes serious safety issues. Although many studies focused on the importance of system transparency in keeping proper trust calibration, the research in detecting and mitigating improper trust calibration remains very limited. To fill these research gaps, we propose a method of adaptive trust calibration that consists of a framework for detecting the inappropriate calibration status by monitoring the user’s reliance behavior and cognitive cues called “trust calibration cues” to prompt the user to reinitiate trust calibration. We evaluated our framework and four types of trust calibration cues in an online experiment using a drone simulator. A total of 116 participants performed pothole inspection tasks by using the drone’s automatic inspection, the reliability of which could fluctuate depending upon the weather conditions. The participants needed to decide whether to rely on automatic inspection or to do the inspection manually. The results showed that adaptively presenting simple cues could significantly promote trust calibration during over-trust.

## Introduction

There are growing interests in automation and autonomous AI technologies in many fields of applications. One highly anticipated application is the unmanned autonomous vehicle, such as driverless shuttles that transport people in rural areas and unmanned aerial vehicles for aerial images, deliveries, and also military purposes. Other expanding application areas such as robotics, autonomous web-based systems, and decision aids are changing all aspects of our daily life.

Collaboration between human users and autonomous AI agents is always essential as such technologies are never perfect. One key aspect of such collaborations is that users trust the agents. Trust is an attitudinal judgment of the degree to which a user can rely on an agent to achieve their goals under conditions of uncertainty [1]. Successful collaborations between users and agents would require the users to appropriately adjust their level of trust with the actual reliability of the agents. This process is called trust calibration [1, 2]. While an agent’s reliability changes for various reasons in an environment, the users often fail to calibrate their trust in the agent and end up in the status called over-trust or under-trust. Over-trust is poorly calibrated trust in which the user overestimates the reliability of the agent; it can result in misuse of an agent that performs outside of its designed capability. Under-trust is poorly calibrated trust in which the user underestimates the agent’s capability; it can result in disuse of the agent, excessive user workload, and/or deterioration of the total system performance. Poor trust calibration sometimes causes serious safety issues [3, 4].

In the current study, we focused on the problem of over-trust or under-trust and propose a novel method of adaptive trust calibration that consists of a framework for detecting the inappropriate status of calibration and cognitive cues called “trust calibration cues” (TCCs) to prompt the user to reinitiate trust calibration.

Considering the challenge of measuring trust, given that it is a complex psychological construct, our framework focuses on trust as observable choice behavior [5] so that we can estimate calibration status by monitoring user behavior. When over-trust or under-trust is detected, a TCC is presented to the user to alert them to recalibrate their trust. Unlike existing studies [6, 7] that emphasize the importance of continuously displaying system information to maintain appropriate trust calibration, our method adaptively presents information that triggers only when it is deemed necessary for users to recalibrate their trust. To our knowledge, most of the existing studies on trust calibration have been about how to prevent inappropriate calibration status; very few have investigated how to mitigate it. The initial idea of the proposed method was discussed in the work [8]. In the current study, we defined a framework and conducted an online experiment with a web-based drone simulator to evaluate the effectiveness of our method in an over-trust scenario. The participants of the experiment performed a pothole inspection task [9] to check if there were any holes or cracks in the road images from the drone. Participants chose to use the drone’s automatic inspection or to check the road image manually. By observing the participants’ choice behavior, the framework judged the trust calibration status and presented TCCs when over-trust was detected. We measured behavioral changes to see if our adaptive method could effectively restore an appropriate status of trust calibration.

We found that adaptively presented TCCs significantly affected the choice behavior of the participants. The participants of the group without TCC did not change their behavior when they were in over-trust status, even if the system reliability information was continuously presented. One of the groups with TCCs had a significantly higher value of sensitivity d’ than the group without TCC, showing that adaptive trust calibration was effective in situations where continuous trust calibration was not effective. Despite several limitations in our framework and our experiment, our study demonstrates the effects of adaptively presenting cognitive cues in instances of over-trust.

In the following subsections, we review related work on trust calibration and propose our adaptive trust calibration approach. Next, we cover our experiment and the results. Finally, we discuss the results and describe our future work.

### Factors influencing trust

Extensive research has been conducted to examine the factors that influence a human’s trust in autonomous agents such as automation. Hancock et al. [10] examined factors that affect trust by applying meta-analysis methods to existing empirical studies. They identified three major trust factors: robot-related factors (performance-based and attribute-based), human-related factors (ability-based and human characteristics), and environment-related factors (team collaboration and task-based factors). They found that the factors related to the robot and its performance had the greatest current association with trust. Hoff and Bashir [11] proposed a three-layered trust model to categorize factors that influence automation trust: dispositional trust representing an individual’s overall tendency to trust, situational trust based on the external environment and context-dependent human characteristics, and learned trust, which is knowledge of a system drawn from past experiences or a current interaction. Learned trust is further divided into two types: initial learned trust, trust prior to interacting with a system, and dynamic learned trust, trust formed during an interaction.

In this study, we investigate trust calibration by focusing on dynamic learned trust factors and performance-based factors such as agent reliability.

### System transparency and continuous trust calibration

Humans require a user interface that captures the state of an entire system in order to interact appropriately with the agent. Studies such as [2, 12, 13] have emphasized the importance of system transparency in maintaining proper trust calibration. System transparency has been defined as “the quality of an interface pertaining to its ability to afford an operator’s comprehension about an intelligent agent’s intent, performance, future plans, and reasoning process [14].” This definition is essentially in accordance with the factors that influence a human’s trust.

McGuirl and Sarter [15] showed how continually updated system confidence information can improve trust calibration and increase the performance of the human-machine team. Studies on visualizing car uncertainty during autonomous driving [16, 17] have indicated that providing good transparency by constantly presenting the system information helps maintain appropriate trust of the vehicles. Seppet [7] demonstrated that continuous feedback on automation behavior viably promotes calibrated trust and reliance.

Most of the studies investigated how to maintain appropriate trust calibration by continuously presenting system information to prevent over-trust or under-trust. Very few studies have focused on detecting poor trust calibration or how to recover from over-trust or under-trust swiftly.

### Measuring trust

Trust is a latent construct and cannot be directly measured; measuring trust experimentally is difficult in general. Most previous literature used self-report measures such as subjective rating with questionnaires [18] or rating scales [19, 20]; however, self-report measures are too intrusive to be viable in applied settings. Physiological and neural measures have been proposed, such as gaze behavior [21], facial and voice tracking [22], heart-rate, and EEG. Although these measures can be used for dynamic tracking of trust, they usually require special hardware.

Behavioral measures are useful for applications in real-world situations. Behavior used as trust measures includes choosing manual or automatic tasks [23], choosing an automation level [24], and accepting advice (reliance behavior). Behaviors might not be observable if there is no interaction; nevertheless, behavioral measures are practical and can easily be used as a basis for modeling and prediction [25].

### Our approach: Adaptive trust calibration

Once users fall into the categories of over-trust or under-trust, it might not be easy for them to escape them. Calibration can only occur in response to new evidence that may change the users’ prevailing recognition, while no new evidence can be learned without changing the current behavior first [5]. To solve this dilemma that could perpetuate an inappropriate status of trust calibration, a new trigger would be necessary in instances of over-trust or under-trust. To realize this idea, we propose a new framework to detect inappropriate trust calibration.

#### Framework for detecting over-trust and under-trust

We propose a framework for detecting the inappropriate status of the trust calibration with a behavior based approach. Suppose a user and an AI agent are jointly working on a set of tasks. The user should decide whether to rely on the agent or do each task manually. In our framework, three parameters, *P*_*auto*_, *P*_*trust*_, and *P*_*man*_, are defined as follows:

- *P*_*auto*_: Probability that a task done by an agent will be successful. This is called the “reliability of the agent.”
- *P*_*trust*_: User’s estimation of *P*_*auto*_. This is a user’s trust in the agent.
- *P*_*man*_: Probability that a task done manually by a user will be successful. This is called the “capability of the user.” Note that “*man*” means “*manual*.”

*P*_*auto*_ varies depending on the conditions of the agent. *P*_*trust*_ also changes accordingly and becomes equal to *P*_*auto*_ if trust is appropriately calibrated. Over-trust occurs if *P*_*trust*_ > *P*_*auto*_, and under-trust occurs if *P*_*trust*_ < *P*_*auto*_. Since measuring *P*_*trust*_ is difficult, we modified the definitions of over-trust and under-trust by using a third parameter *P*_*man*_ in addition to *P*_*trust*_ and *P*_*auto*_ as follows:

- Over-trust: the user estimates that the agent is better at the task than the user even though the actual reliability of the agent is lower than the user’s capability.
  $$\begin{array}{c} \left(P_{trust}>{\hat{P}}_{man}\right)\wedge \left(P_{man}>P_{auto}\right) \end{array}$$ (1)
- Under-trust: the user estimates that they are better at the task than the agent even though the actual reliability of the agent is higher than the user’s capability.
  $$\begin{array}{c} \left(P_{trust}<{\hat{P}}_{man}\right)\wedge \left(P_{man}<P_{auto}\right) \end{array}$$ (2)

${\hat{P}}_{man}$ is a user’s self estimation of *P*_*man*_, which corresponds to the user’s self-confidence. These two parameters were not clearly distinguished in [8]. Several studies [13, 23, 26] have demonstrated that reliance behavior can be explained by the relationship between a user’s trust in the agents and the user’s self-confidence. When a user decides to rely on an agent, it is reasonable to say that this behavior indicates $P_{trust}>{\hat{P}}_{man}$. If the user decides to do the task manually rather than rely on the agent, it indicates $P_{trust}<{\hat{P}}_{man}$. Instead of directly measuring *P*_*trust*_ or ${\hat{P}}_{man}$, the first inequalities of (1) and (2) can be estimated by observing the user’s reliance behavior; thus, the trust calibration status can be detected, if the second inequalities of *P*_*man*_ and *P*_*auto*_ can be estimated.

#### Trust calibration cue as a new trigger

In this study, we explore the novel idea of giving cognitive cues to users when over-trust or under-trust is detected. This cue is expected to trigger the user to promptly notice what has been happening in the environment and to calibrate the trust based on the new findings. We call this cognitive cue a “trust calibration cue” (TCC).

Visser et. al [27] proposed a design guideline for trust cues, which are information elements used to make a trust assessment about an agent. They classified the cues in terms of trust dimensions (intent, performance, process, expressiveness, and origin) and the trust processing stages (perception, comprehension, projection, decision, and execution). Unlike our TCC, their trust cues were to bring the information specific to the dimensions and stages. Komatsu et.al [28] proposed an intuitive notification methodology called “artificial subtle expressions” (ASE). One of the design requirements is “complementary,” which means that notifications should not interfere with the main communication protocol. Cowell et.al [29] discussed the five non-verbal behaviors of an embodied conversational agent. Waytz et.al [30] demonstrated that anthropomorphism increases trust in an autonomous vehicle. Laughert et. al [31] examined three important objectives in effective warnings: attract attention, elicit knowledge, and enable compliance behavior. Based on these pieces of literature, we designed four types of TCCs (Visual/Audio/Verbal/Anthropomorphic) and evaluated them in the experiment. Details will be explained in the next section.

#### Adaptive trust calibration

With the framework and TCCs described above, we propose a method of adaptive trust calibration as follows. (Details of the detection algorithm will be described later).

**Method** Adaptive trust calibration

**loop**

 Observe a user’s reliance behavior on an agent.

 Evaluate expression (1) and (2) of the framework.

 **if** the over-trust or under-trust is detected **then**

  Present a TCC to the user.

 **end if**

**end loop**

The purpose of this method is to adaptively prompt a user to calibrate her/his trust by presenting a TCC only when our framework detects over-trust or under-trust by observing the user’s choice behavior. This approach is to mitigate over-trust or under-trust, in contrast with the traditional approach of trying to maintain appropriate trust calibration with continuous system transparency.

### Hypothesis

We expected users to change their choice behavior if TCCs were adaptively presented when the framework detected inappropriate trust calibration. If our method could effectively mitigate the over-trust or under-trust, the following are hypothesized:

**[H0]** the manual choice rates decrease in cases of over-trust or increase in cases of under-trust.
**[H1]** the users with TCCs perform better than the users without TCCs.

## Materials and methods

All studies were carried out in accordance with the recommendations of the Ethical Guidelines for Medical and Health Research Involving Human Subjects provided by the Ministry of Education, Culture, Sports, Science and Technology and Ministry of Health, Labor, and Welfare in Japan with written informed consent from all participants. All participants gave written informed consent in accordance with the Declaration of Helsinki. The protocol was approved by the ethics committee of the National Institute of Informatics.

### Participants

We recruited participants online through a crowdsourcing service provided by Macromill, Inc. Regarding online experiments in general, Crump et al. [32] showed that the data collected online using a web-browser seemed mostly in line with laboratory results, so long as the experiment methods were solid.

194 participants joined the experiment online. They were between 20 to 69 years old (*M* = 44.35, *SD* = 14.10). 96 participants were male and 98 were female.

### Apparatus and materials

**Fig 1:**
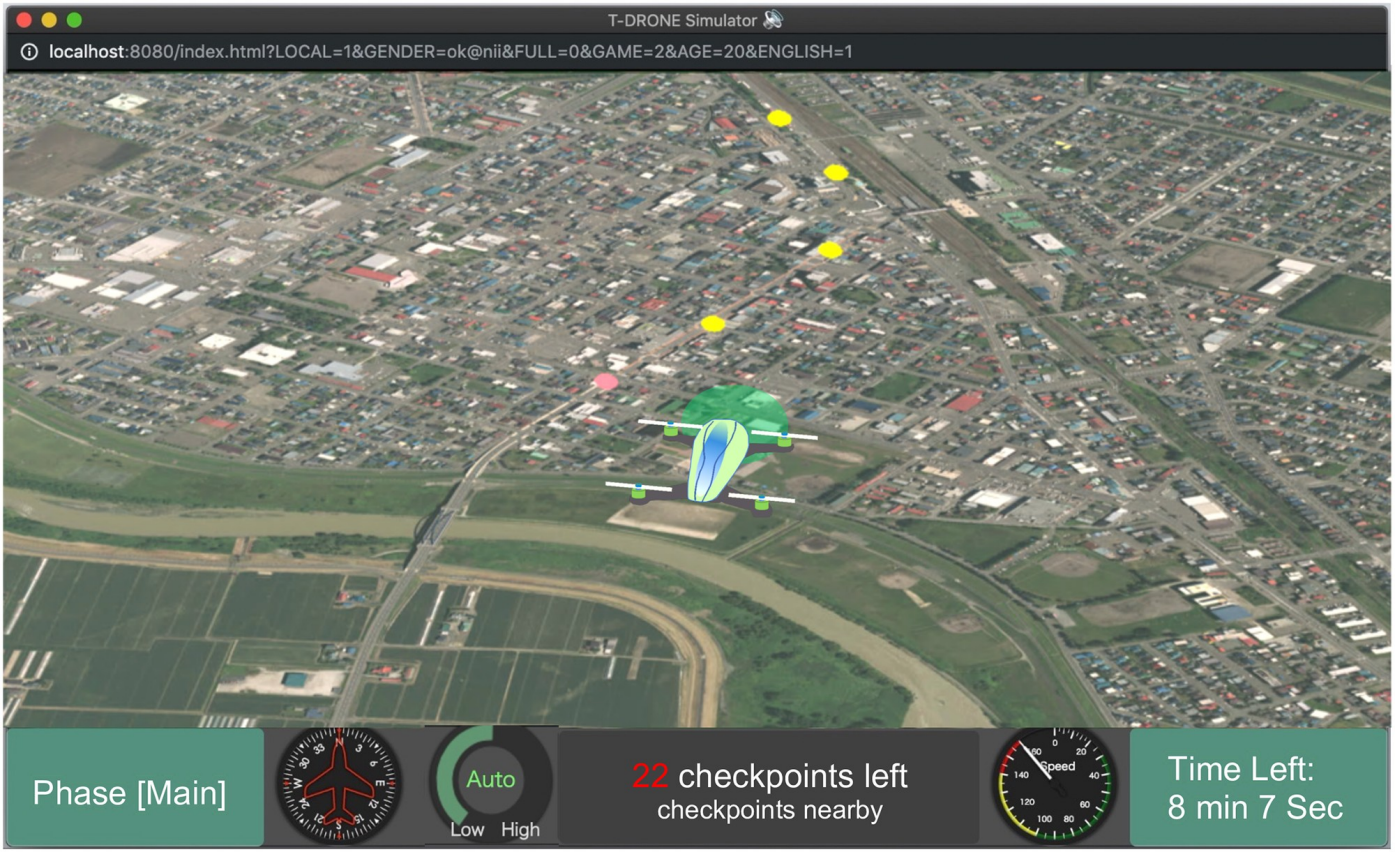
Online drone simulator. Operating the simulator was relatively easy, with two cursor keys for controlling the direction of the drone and mouse buttons for making choices. Geospatial Information Authority of Japan (https://maps.gsi.go.jp) CC BY 4.0. The image is similar but not identical to the original one used in the experiment due to a copyright reason.

We developed a 3D drone simulator based on an open-source JavaScript WebGL library CesiumJS [33] and the Bing Map API [34]. A screenshot of the simulator running on a Chrome browser is shown in Fig 1.

#### Pothole inspection tasks

**Fig 2:**
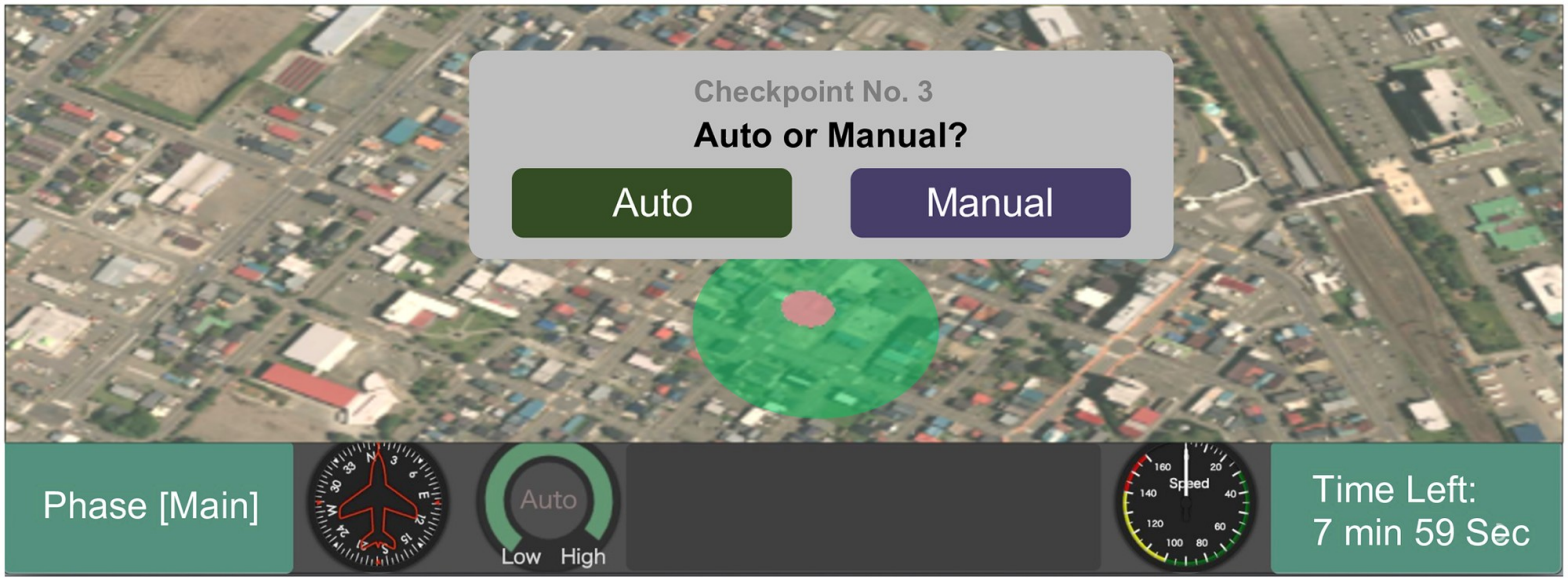
Popup message asking the participants for choice. Geospatial Information Authority of Japan (https://maps.gsi.go.jp) CC BY 4.0 The image is similar but not identical to the original one used in the experiment due to a copyright reason.

A route with 30 checkpoints (CKPs) was defined in the simulated environment. Each CKP was located in the center of a rectangular area that was to be inspected to see if there were any potholes in it. Out of the 30 CKPs, 10 had potholes in the corresponding areas while the other 20 did not. CKPs on the route were shown as small yellow circles on the screen. When the drone came close enough to one of the CKPs on the route, a message popped up (Fig 2) in which the drone asked the participants to make a choice.

**Fig 3:**
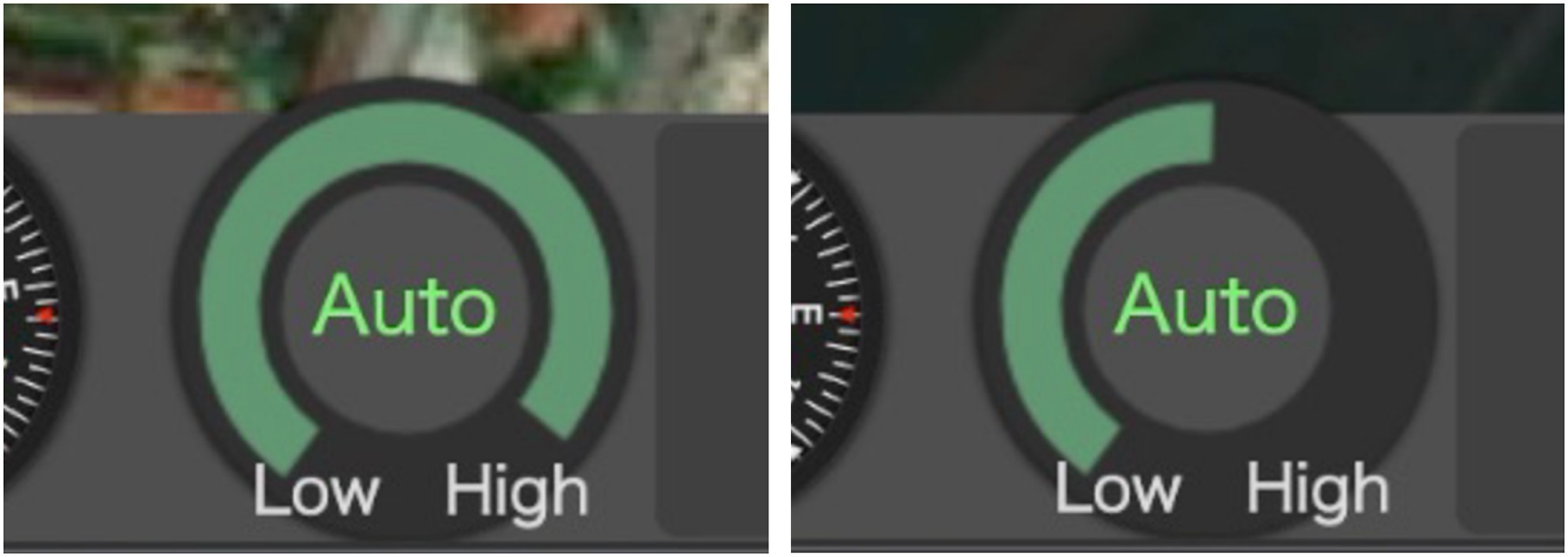
Reliability indicator at the bottom left area of the screen. (A) Showing a higher reliability. (B) Showing a deteriorated reliability.

The indicator at the bottom left area of the screen always showed the reliability of the automatic pothole inspection (Fig 3). This continuous display helped to increase the system transparency in terms of the reliability.

**Fig 4:**
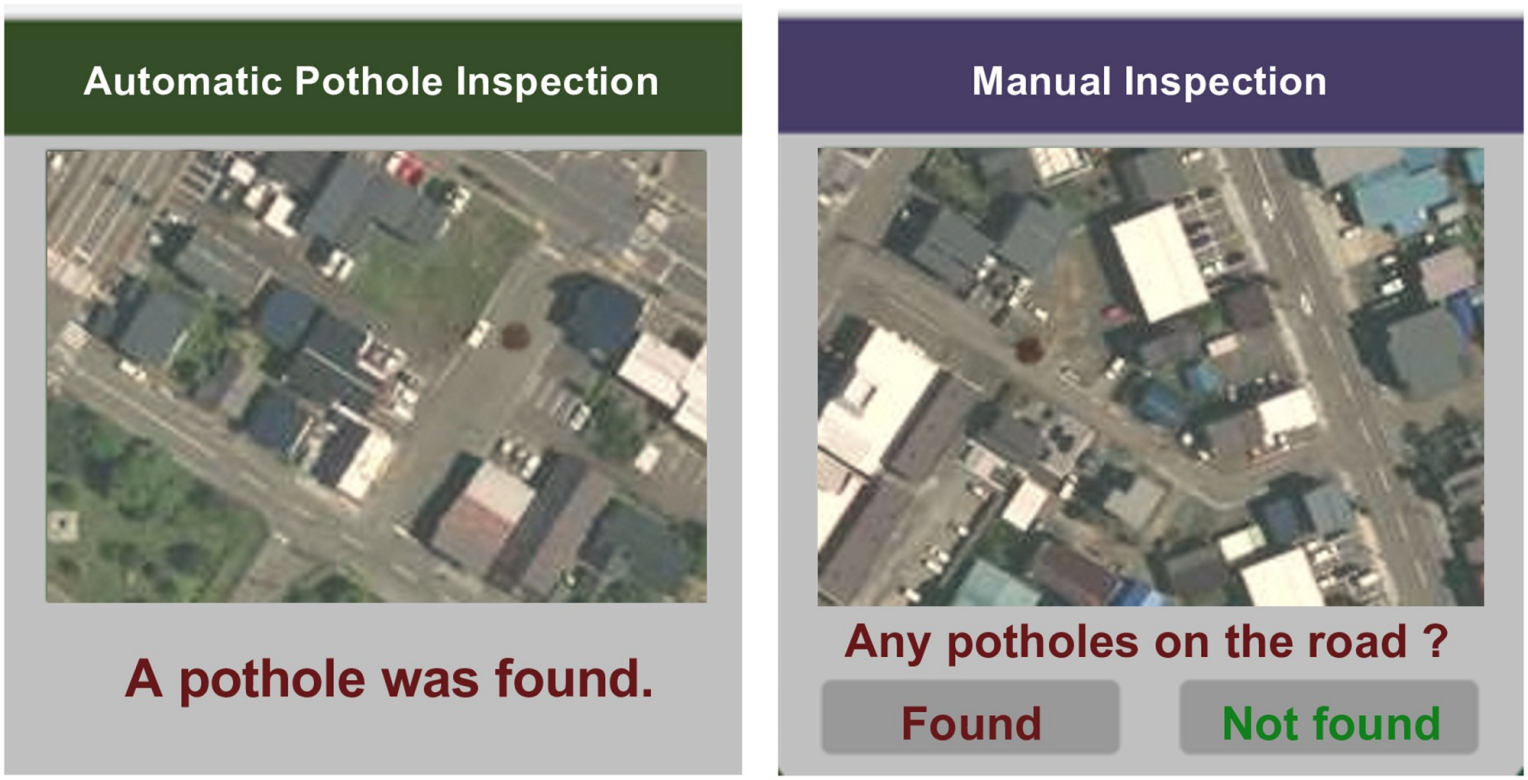
Popup windows of the pothole inspections. (A) Automatic inspection result window. (B) Manual inspection window. Both images contain potholes as dark brown spots in the upper road areas. Geospatial Information Authority of Japan (https://maps.gsi.go.jp) CC BY 4.0. The images are similar but not identical to the original ones used in the experiment due to a copyright reason. Potholes were artificially rendered as irregular shapes in a dark brown color on a road image in the popup window.

If the participants selected the “Auto” button, an automatic-inspection result was shown for three seconds with a road image of the area around the CKP. This feedback information helped the participants understand how well the automatic inspection performed, thereby increasing the system transparency [2, 14]. If the “Manual” button was selected, a road image was displayed, and the participants had to make a pothole report manually. Popup windows of both cases are as shown in Fig 4.

#### Trust calibration cues (TCCs) in experiment

**Fig 5:**
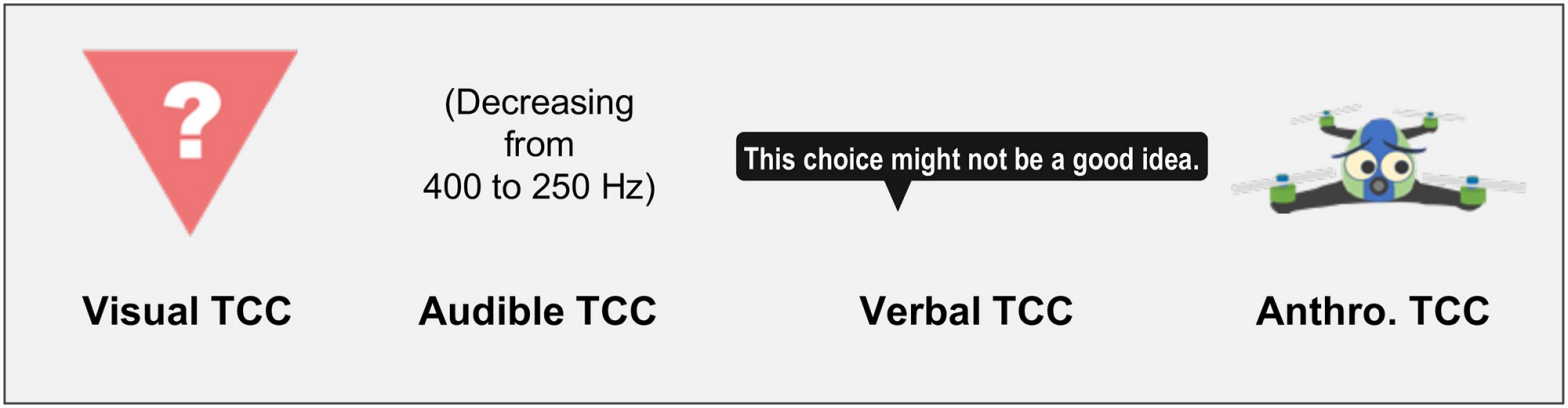
Four types of TCCs. The image of the anthro. TCC is similar but not identical to the original one used in the experiment due to a copyright reason.

We designed and evaluated four types of TCCs (Fig 5) in our experiment.

The visual TCC was designed as a red warning sign in the shape of an upside-down triangle, which is considered to be one of the most common alerting signs according to [31, 35]. The audio TCC uses a sound with a frequency that decreases from 400 Hz to 250 Hz and can convey that the agent has a low confidence level [28]. Verbal TCC is a tooltip balloon with the warning message “This choice might not be a good idea.” The anthropomorphic TCC is an animated drone image with cartoon-like eyes that show the agent’s state. TCCs were presented to the participants when the framework detected the over-trust status: the audio TCC was played once, and other TCCs were displayed on the screen close to the “Auto” button for two seconds.

### Over-trust detection based on the proposed framework

The following algorithm was used to detect over-trust. Note that a simple moving average of three CKP window was used here.

**Algorithm** Over-trust detection

**Initialize**:

Total number of check points(CKPs): M = the number of CKPs.;

Over-trust flag list: OT[1], …, OT[M] are initialized with zero;

The number of current CKP: *i* ⇐ 1;

**while** *i* ≦ *M* not time-over **do**

 **if** the drone reached a CKP **then**

  **if** choice behavior is AUTO **and** *Pman* > *Pauto* **then**

   *OT*[*i*] ⇐ 1;

   **if** *i* ≧ 3 **and** (*OT*[*i* − 2] + *OT*[*i* − 1]) ≧ 1 **then**

    Over-trust is detected and TCC is presented to the user;

   **end if**

  **end if**

  *i* ⇐ *i* + 1;

 **end if**

**end while**

### Manipulation of *P*_*auto*_

The parameter *P*_*auto*_ was manipulated to evaluate our method in an over-trust scenario. The performance of the automatic pothole inspection was configured based on signal detection theory(SDT) [36]. The SDT describes the detection of signals in noisy environments. The noise and the signal are represented as two overlapping density distributions. The distance between the two curves represents the sensitivity *d*′ of the system.

**Fig 6:**
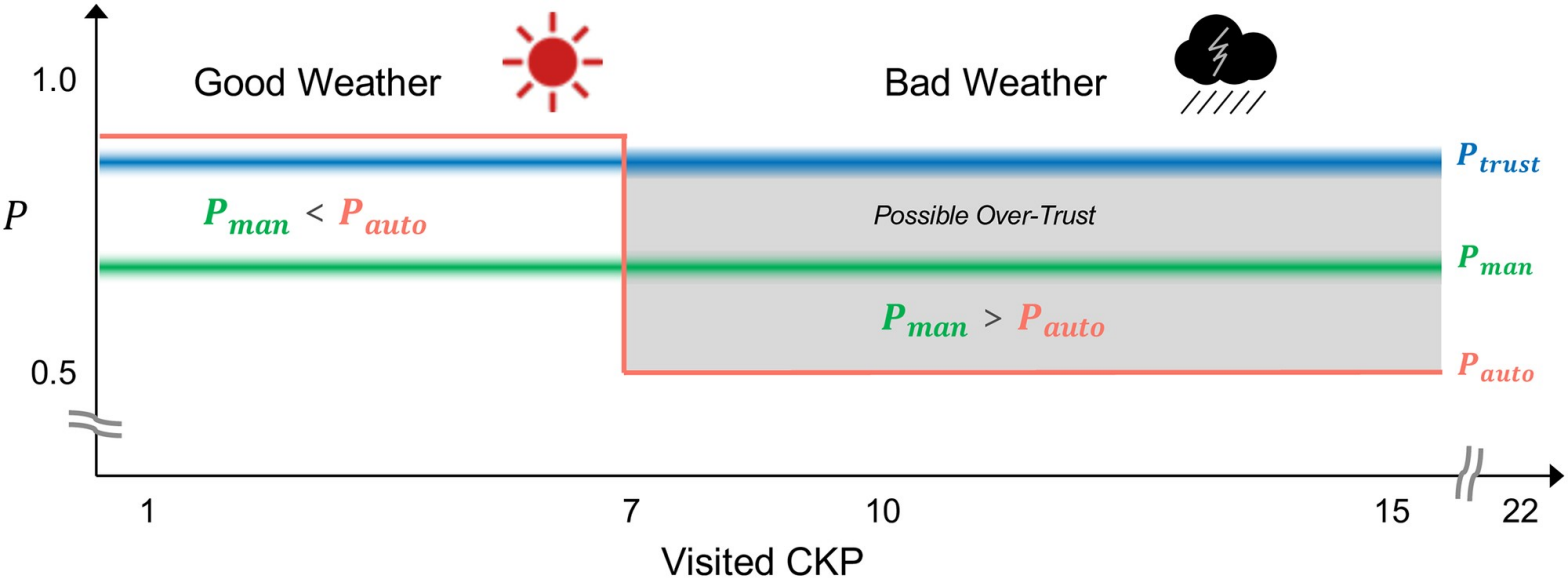
Relationship between the three parameters under changing weather conditions.

In this experiment, the underlying base rate of potholes was 0.3. Under good weather conditions, *P*_*auto*_ and the corresponding sensitivity *d*′ were 90% and 1.8 respectively, indicating a pretty good discriminating ability of the agent. Under bad weather conditions, *P*_*auto*_ dropped to 50% and the corresponding sensitivity *d*′ became 0.1, meaning the reliability of the automatic pothole inspection had greatly deteriorated. Fig 6 illustrates the manipulation of *P*_*auto*_ and its relationship with *P*_*trust*_ and *P*_*man*_.

Participants were expected to reach at least the 15th CKP in this experiment.

### Assumptions

The images of the potholes were carefully designed so that the average success rate of manual inspection would be more than 75%. Although machine image recognition has been remarkably advanced with deep neural networks [37], Geirhos et al. [38] and Dodge et al. [39] demonstrated that human object recognition outperforms the top-performing deep neural networks under image degradation, such as Gaussian blur and additive Gaussian noise. These findings could be applied to the estimation of the inequation of *P*_*man*_ and *P*_*auto*_ in the experiment because the pothole inspection became an image recognition task with dark and foggy road images when the weather conditions turned worse. Therefore, we assumed that *P*_*auto*_ would fluctuate more widely than *P*_*man*_ under changing weather conditions. On the basis of this assumption, we calculated the inequality relationship between *P*_*auto*_ and *P*_*man*_ in the experiment; the inequality *P*_*auto*_ > *P*_*man*_ was true during the good weather period and false during the bad one.

### Procedure

Participants were randomly assigned to one of five groups with the corresponding TCCs: NoTCC group (without TCCs), visual group (with the visual sign TCC), audio group (with the audio TCC), verbal group (with the verbal TCC), and anthro. group (with the anthropomorphic TCC). The NoTCC group was the control group in this experiment.

The experiment was completed online in three phases. In **the instruction phase**, the participants were given instructions stating the goal of the experiment was to inspect 22 CKPs out of 30 CKPs on a test route within a time limit. The participants learned they could inspect CKPs by checking the road image manually or by relying on the drone’s automatic pothole inspection capability. They were told that the average success rate of manual pothole inspection was 75% so that they could adjust their initial self-confidence ${\hat{P}}_{man}$ accordingly. They also learned that the reliability of the automatic pothole inspection was very high, although it could fluctuate depending on the conditions of the weather and sunshine. At the end of the instructions, the participants were guided to adjust the sound volume level by listening to a 400 Hz beep sound. Next, in **the training phase**, the participants started to fly the simulated drone in the training mode. They learned how to operate the drone and how to inspect the CKPs with on-screen guides. When the first three CKPs were inspected, the training mode was finished and **the main phase** was started. The reliability of the drone’s pothole inspection *P*_*auto*_ was artificially manipulated by changing the conditions of the weather and sunshine in the simulated environment. Initially, the weather was good, and *P*_*auto*_ was set to 90%. The fine weather continued until the drone visited six CKPs in the main phase. This period of six CKPs was intended for the participants to calibrate their trust toward the drone with a higher reliability of automatic inspection under the good weather conditions. Immediately after the 6th CKP was inspected, sounds of a thunderstorm began. The visibility of the field also became very low, and the *P*_*auto*_ was decreased from 90% to 50%, which changed the sign of *P*_*man*_ − *P*_*auto*_. During this period, the participants were expected to over-trust the drone due to carry-over from the previous weather condition. The proposed framework was evaluated in this period. When the participants clicked the “Auto” button for automatic inspection and the framework detected the over-trust status using the over-trust detection algorithm described above, the corresponding TCC was presented at the timing right after the button was selected. The following information was available to the participants during the experiment:

- Simulated weather information with visibility changes, brightness changes, and the sounds of a thunderstorm.
- A reliability indicator to continuously show the reliability of the automatic inspection.
- An enlarged photo images of each CKP.
- The result of the drone’s automatic inspection when AUTO was chosen.
- A TCC when the proposed framework detected over-trust.

The experiment ended if the 22nd CKP was inspected or the time limit was reached. We established a time limit of 8.5 minutes (510 seconds) based on pre-trials with this test route, and we expected a single automatic inspection to take 10 seconds, one manual inspection to take 15 seconds, and reaching the next CKP to take 10 seconds.

### Dependent measures

The dependent variables of interest in this experiment were a TCC presentation rate(hereinafter, called TCC rate), a manual choice rate(hereinafter, called manual rate), sensitivity *d*′, and accuracy of the task results. TCC rates mean how often the proposed framework detected over-trust. Changes in manual choice rates indicate how the participants changed their behaviors as a result of trust calibration. Both sensitivity *d*′ and accuracy indicate the performance of the human-AI collaboration.

All keyboard inputs and mouse clicks were recorded and used to calculate these variables.

## Results

One hundred sixteen participants successfully inspected 15 CKPs or more within the time limit. Seventy eight participants unintentionally moved the drone far from the area where the CKPs were located, and they failed to complete the tasks within the time limit. As for the successful participants, their ages ranged from 20 to 69 years old (*M* = 43.25, *SD* = 14.01), 66 participants were male and 50 were females. 28 were in the NoTCC group, 18 in the visual group, 22 in the audio group, 29 in the verbal group, and 19 in the anthro. group. They inspected the total of 1,740 CKPs from the 1st CKP to the 15th CKP, and the results of 1,282 inspections were correct, making the correct answer rate 0.74. Automatic inspection was selected 1,236 times (the choice rate = 0.71). The participants did the manual inspection 504 times (the choice rate = 0.29). Table 1 shows the TCC rates at each CKP. Note that TCCs were not presented in the period from the 7th CKP to the 9th CKP, since the sliding window of three CKPs was used in the detection algorithm. Means and standard errors of the other dependent measures can be found in Table 2. Hereinafter, we call the period from the 1st visited CKP to 6th CKP “the good weather period” and the period with possible TCC presentations from the 10th CKP to 15th CKP “the bad weather period”.

**Table 1:** Means of TCC rates at each CKP.

group	CKP9	CKP10	CKP11	CKP12	CKP13	CKP14	CKP15
Visual TCC	0.78 (0.10)	0.67 (0.11)	0.56 (0.12)	0.67 (0.11)	0.67 (0.11)	0.50 (0.12)	0.56 (0.12)
Audio TCC	0.55 (0.11)	0.64 (0.10)	0.45 (0.11)	0.50 (0.11)	0.50 (0.11)	0.50 (0.11)	0.50 (0.11)
Verbal TCC	0.48 (0.09)	0.45 (0.09)	0.28 (0.08)	0.31 (0.09)	0.34 (0.09)	0.38 (0.09)	0.07 (0.05)
Anthro TCC	0.53 (0.11)	0.47 (0.11)	0.37 (0.11)	0.53 (0.11)	0.47 (0.11)	0.74 (0.10)	0.63 (0.11)

Standard errors in parentheses.

**Table 2:** Means of the other dependent measures.

Group	Manual rate		Sensitivity d’		Accuracy	
	Good weather	Bad weather	Good weather	Bad weather	Good weather	Bad weather
NoTCC	0.15 (0.04)	0.22 (0.06)	1.36 (0.09)	0.38 (0.14)	0.86 (0.04)	0.60 (0.04)
Visual	0.11 (0.06)	0.37 (0.09)	1.35 (0.13)	0.52 (0.15)	0.87 (0.05)	0.62 (0.04)
Audio	0.20 (0.06)	0.42 (0.08)	1.62 (0.10)	0.61 (0.15)	0.94 (0.03)	0.65 (0.04)
Verbal	0.17 (0.05)	0.63 (0.04)	1.39 (0.10)	0.92 (0.14)	0.87 (0.04)	0.72 (0.04)
Anthro.	0.20 (0.06)	0.37 (0.07)	1.40 (0.15)	0.54 (0.15)	0.88 (0.05)	0.62 (0.04)

Standard errors in parentheses.

“Good weather” means the good weather period and “Bad weather” means the bad weather period.

### TCC rates

TCC rates in the verbal TCC group were higher in the early part of the period and gradually decreased. This indicates that over-trust decreased during this period. The visual and audio TCC groups also showed a similar trend, while TCC rates in the anthro. TCC group did not follow the decreasing trend.

### Manual rates

TCCs were presented multiple times per participant in most cases. The effects of presenting TCCs might be accumulated and did not always appear immediately after presentation. In the current study, we evaluated the TCC effects by comparing the six-CKP mean values of the manual rates both for the good and bad weather periods, so that we could also capture the accumulated effects in each period. We conducted a two factor mixed ANOVA with the TCC groups (NoTCC, visual, audio, verbal, and anthro.) as between subjects and CKP periods (the good weather period and the bad weather period) as within subjects. The analysis revealed a significant main effect for the CKP periods [*F*(1, 111) = 51.69, *p* < 0.01, $\eta_{p}^{2}=0.32$]. The participants changed their choice behavior as the weather conditions deteriorated. A significant interaction was found between the two factors [*F*(4, 111) = 4.86, *p* < 0.01, $\eta_{p}^{2}=0.15$].

**Fig 7:**
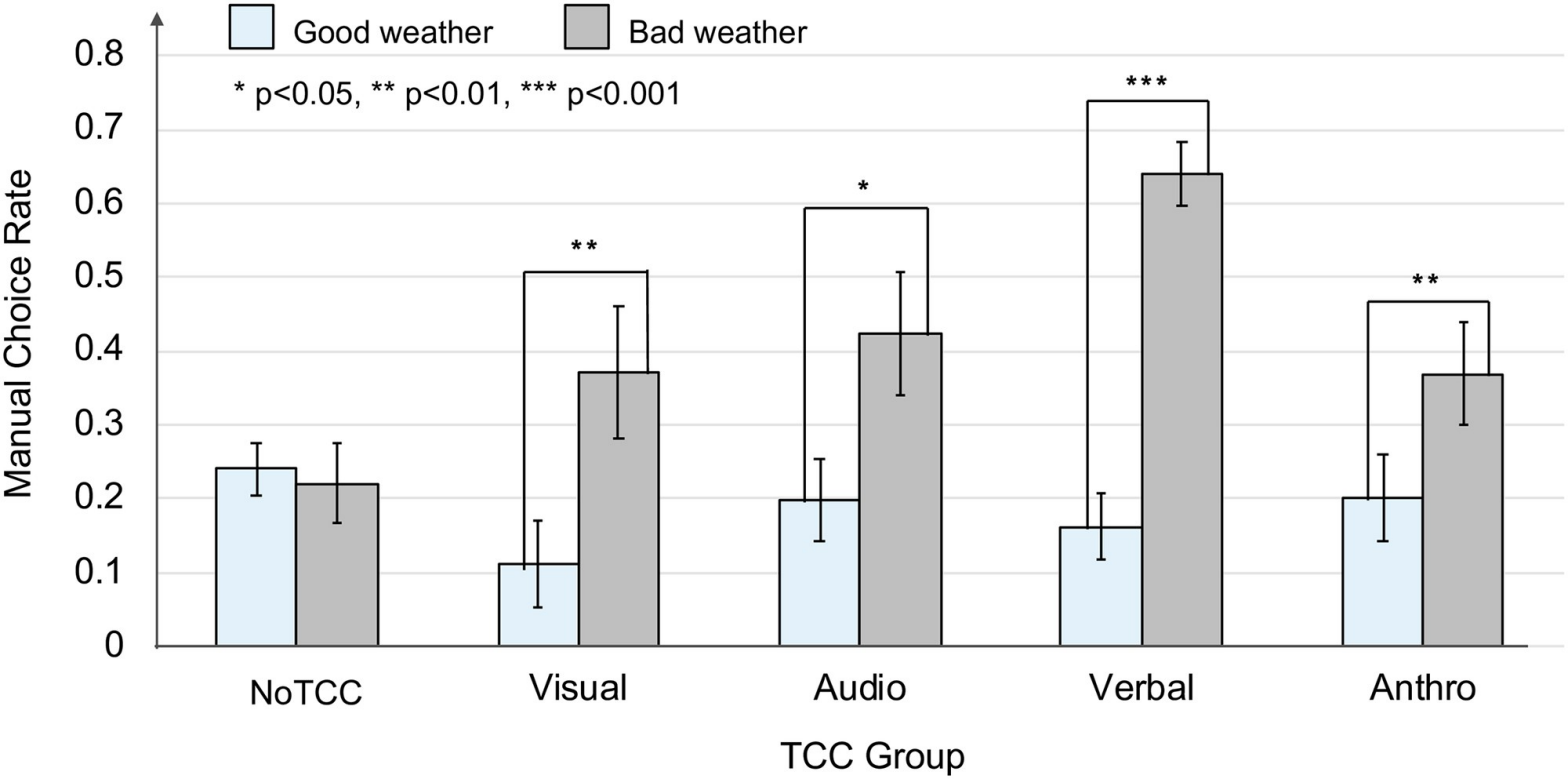
Manual rates over time.

In the good weather period, there was no simple effect for the TCC groups, meaning that the manual rates of each TCC group were not significantly different from each other [$F\left(4,111\right)=0.47,p=0.76,\eta_{p}^{2}=0.02$]. The NoTCC group did not show a simple effect for the CKP periods [$F\left(1,27\right)=1.23,p=0.28,\eta_{p}^{2}=0.04$] indicating the manual rates of the NoTCC group were not significantly different between the two CKP periods. In contrast with this, all of the groups with TCCs showed significantly higher manual rates in the bad weather period than in the good weather period [Visual TCC, *F*(1, 17) = 9.20, *p* < 0.01, $\eta_{p}^{2}=0.35$; Audio TCC, *F*(1, 21) = 5.54, *p* = 0.03, $\eta_{p}^{2}=0.21$; Verbal TCC, *F*(1, 28) = 62.9, *p* < 0.001, $\eta_{p}^{2}=0.69$; Anthro TCC, *F*(1, 18) = 8.55, *p* < 0.01, $\eta_{p}^{2}=0.32$]. Fig 7 shows how the manual rates changed over two CKP periods.

Holm–Bonferroni-adjusted post hoc comparisons were also conducted to investigate the effects of TCCs. For the bad weather period, the verbal group showed a significantly higher manual rate than both the NoTCC group (*t*(111) = 4.77, *adj*.*p* < 0.01) and the anthro. group (*t*(111) = 2.74, *adj*.*p* = 0.04). No other differences between the groups were found to be significant. Although the effectiveness among TCCs differ, these results support **H0**.

### Performance: Sensitivity *d*′ and accuracy

The same ANOVA with the TCC groups and the CKP periods revealed that the sensitivity *d*′ in the bad weather period was significantly lower than in the good weather period [$F\left(1,111\right)=107.22,p<0.01,\eta_{p}^{2}=0.49$]. In the bad weather period, post-hoc comparisons indicated that the sensitivity *d*′ of the verbal group, which was the highest (0.92 (SE 0.14) among all the groups, was significantly higher than the sensitivity *d*′ of the NoTCC group [*t*(111) = 2.97, *adj*.*p* = 0.04, *Cohen*’*s* *d* = 0.75]. In terms of sensitivity *d*′, hypothesis **H1** is supported.

Accuracy, the rate of the correct inspection, also significantly declined in the bad weather period[$F\left(1,111\right)=87.58,p<0.01,\eta_{p}^{2}=0.46$], but there was no significant difference among the five groups[$F\left(4,111\right)=1.62,p=0.18,\eta_{p}^{2}=0.06$]. Hypothesis **H1** is not supported regarding accuracy; however, the verbal group showed the highest mean value (0.72 (SE 0.04)), and other groups with TCCs also had better accuracy values (0.6 (SE 0.04)) than the NoTCC group.

Regarding the accuracy of the manual inspections which corresponds to *P*_*man*_, Table 3 shows the 3-CKP mean values of *P*_*man*_. Although the mean values of *P*_*man*_ slightly increased, a Welch’s t-test indicated that there was no significant difference between *P*_*man*_ in the good weather period and in the bad one [*t*(114) = −1.08, *p* = 0.28, *Cohen*’*s* *d* = 0.16]. This result indicates that *P*_*man*_ did not degrade under the change in weather conditions. One-sample t-tests showed that *P*_*man*_ in the good weather period was significantly smaller than *P*_*auto*_ = 0.90 [*Mean* = 0.81, *t*(71) = −2.17, *p* = 0.03, *Cohen*’*s* *d* = 0.26] and that *P*_*man*_ in the bad weather period was significantly larger than *P*_*auto*_ = 0.50 [*Mean* = 0.86, *t*(200) = 17.79, *p* < 0.01, *Cohen*’*s* *d* = 1.25].

**Table 3:** 3-CKP mean values of *P*_*man*_.

CKPs	Good weather period		Bad weather period		
	1 to 3	4 to 6	7 to 9	10 to 12	13 to 15
*P*_*man*_	0.81 (0.05)	0.80 (0.06)	0.85 (0.04)	0.86 (0.03)	0.90 (0.03)

Standard errors in parentheses.

## Discussion

The overall results demonstrated that adaptively presenting TCCs strongly affected whether the choice behavior of the participants would change, while continuously presenting the reliability information did not help the participants change their bias to rely on the automation. The better task performances were also achieved with the behavior changes triggered by TCCs, whose presentation timing was decided by the proposed framework.

### Effects of TCCs to change the participants’ behavior

**Fig 8:**
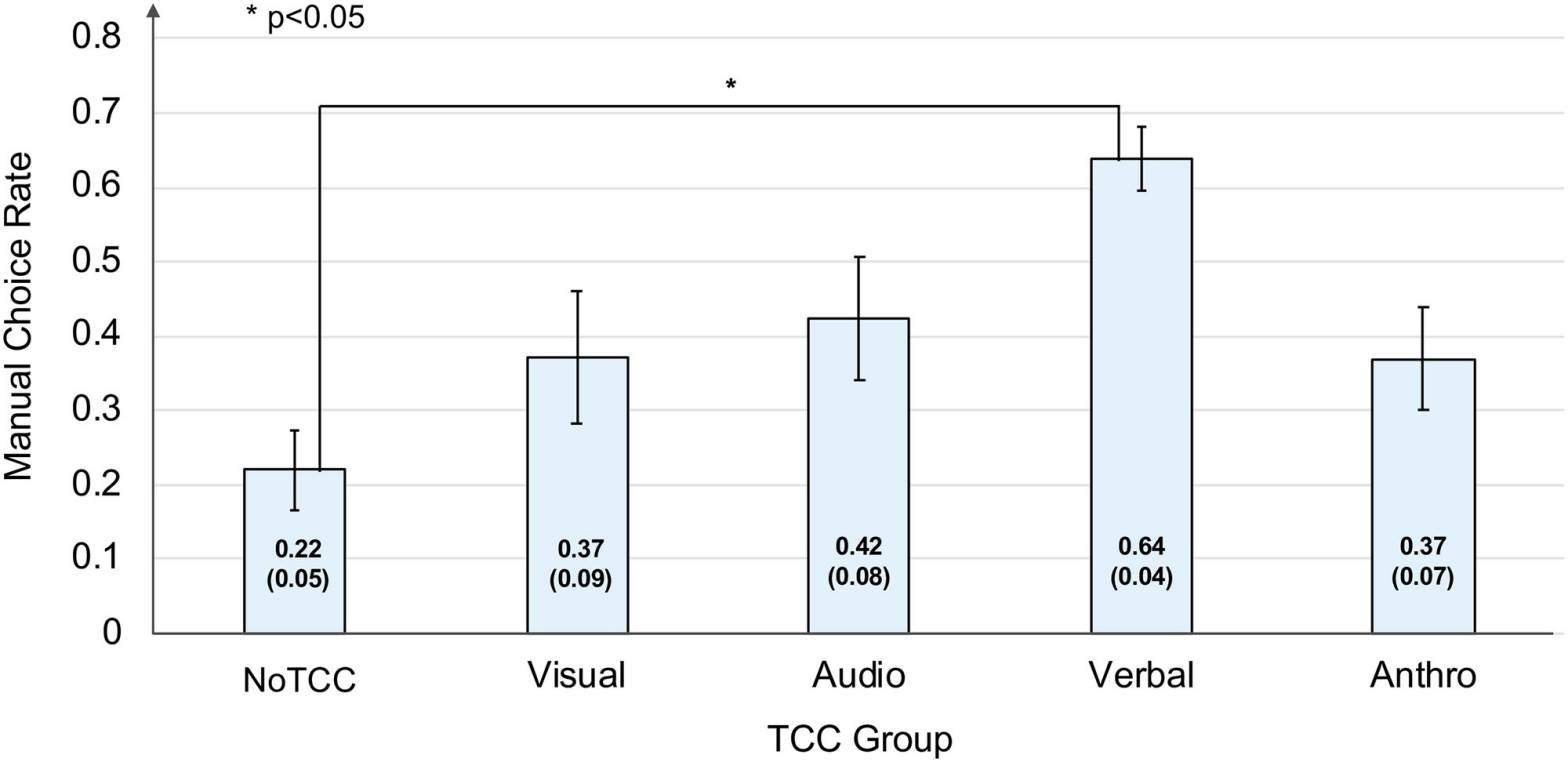
Manual rates for each TCC during the bad weather period.

The groups with visual, verbal, and anthro. TCCs showed significantly higher manual rates for the bad weather period than for the good weather period (Fig 7). The audio TCC that used an audio ASE that decreased in frequency seemed promising, as it could convey the low level of confidence in the system [28]. The result, however, showed that its manual choice rate was not significantly larger than the one of NoTCc group. One possible reason is that other sounds were also being played in the simulated environment, such as the drone flying and the thunderstorm, which may have reduced the effect of this audio TCC. The effect of the anthro. TCC was not as large as we originally expected; it was significantly smaller than the effect of the verbal TCC (Fig 8). The manual choice rate of the visual TCC were also smaller than other TCCs. It was obvious that the participants recognized these visually impressive TCCs; however, the results suggested that just being salient on the screen was not enough for some of the participants to change their choice behavior [31, 40]. The verbal group had the highest manual rate (Fig 8). Only the verbal TCC referred to the purpose by using the word “choice,” while the other TCCs were implemented just as a caution or warning. Reading this word might have helped the participants proceed more easily to the latter stages of the trust calibration process.

Based on these results, our tentative guideline for designing TCCs is that TCCs should be reasonably noticeable in the task environment and should contain connotations that can link the user to the next possible actions in the collaborative task.

If the participants wanted to complete the scan tasks quickly, they could have used the automatic inspection, which was faster. However, the participants promptly increased the manual choices after recognizing TCCs despite the longer completion time. This result indicates that the possible automation bias caused by the difference in the task completion times did not critically impact the decision-making of the participants in the experiment.

These results indicated that adaptively presenting TCCs strongly affected the choice behavior of the participants who otherwise failed to find opportunities to change their tendency to rely on the automation.

### Performance

The results of the manual accuracy were consistent with our assumption to estimate *P*_*man*_ − *P*_*auto*_ in this experiment.

While the mean values of sensitivity *d*′ in each group were dropped in the bad weather period, the verbal group showed a significantly higher sensitivity *d*′ than the NoTCC group, and three other groups with TCCs also had better values than the NoTCC group. The results showed that all the groups with TCCs showed higher discriminating performance in the bad weather period than the NoTCC group. Though there was no statistical difference in accuracy among the groups, all the groups with TCCs also showed better accuracy than the NoTCC group.

These results indicated that adaptively presenting TCCs promoted appropriate trust calibration leading to the better performance in the bad weather period.

### Adaptive method vs. continuous method

The manual rate of the NoTCC group did not significantly change over the two periods. When the participants were exposed to the bad weather, the weather change was made very noticeable with the screen visibility and the sound effects. The reliability indicator showed a big performance degradation of the system due to the poor visibility. Nevertheless, the participants of the NoTCC group continued to rely on the drone’s automatic pothole inspection, which had less reliability than the actual manual success rate. Thus, the participants over-trusted the automatic inspection despite the system information indicating the reliability becoming worse. This result is not in line with the previous studies [6, 7, 15] that emphasized the effectiveness of the continuous trust calibration with system transparency. A possible explanation for the result could be made by discussing models for the trust process [1, 27, 41]. Miring et al.[42] defined a model with four stages: perception, understanding, prediction, and adaption. Although the reliability indicator continuously displayed the deterioration of the reliability, the participants in the NoTCC group might not fully acquire the knowledge to move beyond the perception stage. They would have behave differently if the experiment had continued longer enough for them to understand the relationship between the indicator change and the performance of the system. In contrast to this, the participants in other groups with TCCs could successfully reached the adaptation stage and change their behaviors in this experiment. We believe that the results demonstrated the effectiveness of the adaptive method. TCCs were given right after the behavior only if the participants were judged to over-trust, so that it would be easier for the participants to understand the implication of the cues and to move forward in the trust calibration process.

### Applicability in real-world situations

Although providing a model to estimate the second inequalities of *P*_*man*_ and *P*_*auto*_ in the proposed framework is beyond the scope of this paper, we believe that they could be estimated as follows. *P*_*auto*_, which represents the reliability of an AI agent, could be calculated with the sensor models and algorithms used to implement the AI agent. *P*_*man*_, which is a human capability index, could be estimated by using the parameters of a target task and environmental conditions. The results of the previous studies [38, 39] are such examples that provide a basis for estimating the second inequalities. If an appropriate estimation model for *P*_*man*_ is not available, trial operations can be performed to collect the necessary data to estimate *P*_*man*_ empirically. In practical situations, it is quite common for users of a system to practice how to operate the system in advance. The second inequalities could be estimated even in a real-time situation. The first inequalities in the proposed framework could be estimated by observing users’ choice behaviors, without measuring *P*_*trust*_ and ${\hat{P}}_{man}$ directly. Although a pop-up dialogue was used to observe the behaviors in the current experiment, continuous measurements of the behaviors could also be used with the proposed framework. For example, a driver’s intention to use automatic driving could be inferred with a touch sensor on a steering wheel to check if the driver’s hands are on the wheel. Similarly, a switch button to turn automation on and off at any time could provide necessary information on humans’ reliance on the automation. The first inequality in the framework could be calculated with the information from these continuous methods that could work well with real-time tasks. Therefore, we believe that our proposed framework can be applied to real-time applications that require human-agent collaboration.

### Limitations and future research

The several limitations of our study suggest the need for further experiments and future research.

The current framework focuses on performance-related factors to detect over-trust and under-trust. The capabilities of humans and agents are compared to identify the possible choice behavior that might lead to a better performance. However, automation could be beneficial beyond a better performance, such as for faster task completion, lighter workloads, fewer risks, etc. [3] Therefore, trust as the observable and rational choice behavior can be a product of believing that these benefits will outweigh the costs [5]. For example, Naujoks et.al [43] discussed the desire to engage the non-driving-related tasks during autonomous driving, which requires the workload of the driving task to be lighter and makes the driver select the autonomous mode. Our framework could be integrated with such factors by adjusting the effect of the term *P*_*man*_ with a coefficient *γ*, which represents a degree of human intention to do the main task manually.

$$\begin{array}{c} P_{man}^{\prime }=\gamma *P_{man} \end{array}$$ (3)

For example, if human would not do a task manually because they want to continue a secondary task, a value of *γ* < 1 indicates a negative bias toward the manual choice. Similarly, a value of *γ* > 1 represents a positive bias in doing a task manually. *γ* can be decided in a practical manner by experimenting with several levels of pre-defined factors that influence auto-manual choice decisions. Dual-task paradigm [44] could be applied to determine the values of *γ*.

The feedback information given for each inspection was very important for the participants to make decisions. The pothole inspection task in the experiment is a remote sensing task, and it would be quite difficult for the system to know the correct answer (ground truth) at the time of each inspection in practical situations because the only information available is the image data and the results of automatic recognition. Therefore the correct answer for each inspection was not presented to the participants in the experiment. The result of the automatic inspection was shown to the participants when they selected automatic inspection, not when they did the inspections manually. Although this was to simplify the conditions and focus on evaluating the effect of presenting TCCs, further study should consider possible combinations of feedback information and evaluate their effects.

In this experiment, we focused on evaluating an over-trust case, which often has more serious adverse effects in actual situations [45, 46]. Experiments are currently being prepared with bi-directional trust change scenarios to evaluate cases of over-trust and under-trust. The proposed method deals with users’ behaviors to choose Auto or Manual and does not guarantee the convergence of the behaviors, which is the result of trust calibration by users. Future research could investigate a better way to develop the algorithm in light of control theory, where the trust status would be represented as a non-binary variable, and TCCs could also be presented in a different way.

We used a pothole inspection task in the experiment, which is often categorized as a reconnaissance task in the trust research literature [13]. Future research should explore different types of tasks, such as autonomous driving, decision aids, and interactive games.

Further experiments should be to evaluate our approach with the different types of TCCs to investigate the requirements of effective cues. The current study mainly dealt with dynamic learned trust [11]. Future studies should investigate other factors of trust such as dispositional trust [1] and situational trust to gain a deeper understanding of trust calibration.

## Conclusion

To our knowledge, this is the first study to show how to adaptively prompt the users to calibrate their trust toward an automated agent based on their trust calibration status. Previous studies emphasized the importance of the system transparency for proper trust calibration. Our results indicated that they are not always sufficient to recover from over-trust, and our method of adaptive trust calibration significantly helped the participants change their behavior and recover from the over-trust. Despite several limitations, this study has demonstrated the effectiveness of presenting cognitive cues at the time of over-trust. We strongly believe that the findings of this study will contribute to better user interface designs for collaborative systems with autonomous AI agents.

## Supporting information

S1 File Complete data set. https://doi.org/10.6084/m9.figshare.11538792.v1.
(XLSX)
